# Management of cervical cancer patients during the COVID-19 pandemic: a challenge for developing countries

**DOI:** 10.3332/ecancer.2020.1060

**Published:** 2020-06-17

**Authors:** Maria del Pilar Estevez-Diz, Renata Colombo Bonadio, Vanessa Costa Miranda, Jesus Paula Carvalho

**Affiliations:** 1 Instituto do Cancer do Estado de Sao Paulo—Faculdade de Medicina da Universidade de Sao Paulo, Sao Paulo 01246-000 2, Brazil; 2 Oncologia D’Or, Sao Paulo 04501-000, Brazil

**Keywords:** COVID-19, coronavirus, pandemic, cervical cancer, developing countries

## Abstract

During the COVID-19 pandemic, health services worldwide are going through important adaptations to assist patients infected with COVID-19, at the same time as continuing to provide assistance to other potentially life-threatening diseases. Although patients with cancer may be at increased risk for severe events related to COVID-19 infection, their oncologic treatments frequently cannot be delayed for long periods without jeopardising oncologic outcomes. Considering this, a careful consideration for treatment management of different malignancies is required.

Cervical cancer is concentrated mainly in low-middle income countries (LMICs), which face particular challenges during the COVID-19 pandemic due to the scarcity of health resources in many places. Although cervical cancer is the fourth cause of cancer death among women, it receives little attention from international Oncology societies and scientific research studies. In this review paper, we discuss the cervical cancer landscape and provide specialists recommendations for its management during the COVID-19 pandemic, particularly focused on LMICs’ reality.

## Introduction

Since December 2019, the outbreak of a new coronavirus, the SARS-COV-2 (COVID-19) has been observed with a fast spread worldwide. Currently, countries from all over the world are dealing with the consequences of the COVID-19 pandemic. By the end of May 2020, more than 340,000 fatal COVID-19 cases have been registered and numbers continue to rise exponentially [1]. Facing this, governments have been adopting incisive strategies to minimise the number of individuals with COVID-19 infection and prepare health facilities to assist these cases.

An increased risk of complications from COVID-19 infection has been observed in certain groups such as older patients and those with chronic diseases. Regarding patients with cancer, the data available suggest higher rates of severe events. In a prospective Chinese cohort, among 1506 patients with acute respiratory symptoms with confirmed COVID-19 infection who were hospitalised, 18 patients had a history of cancer. Despite the small sample size and its heterogeneity in terms of primary tumour and phase of treatment, the study suggested that patients with cancer history had 3.56 times (95% CI 1.8–16.1) higher rates of severe events in comparison with those without cancer [2]. In another study with 28 COVID-19 infected cancer patients, receiving oncologic treatment in the last 14 days previous to infection was identified as a risk factor for severe events (HR 4.07, 95% CI 1.08–15.3) [3].

The presence of an active malignancy and the oncologic treatment can lead to the impairment of physical capacity (performance-status) and immunosuppressive states and can increase the requirement for health service visits and hospitalisation [4]. All these factors may contribute to the increased risk of COVID-19 infection and the occurrence of severe events. Considering this, as well as the global efforts to minimise the overwhelming of health services in general, many cancer centres and oncology societies have been discussing the need for clinic visits and oncologic treatment procedures in different scenarios; however, many malignancies represent a considerable threat to patients’ lives and treatment delays may impact oncologic outcomes. Thus, management recommendations should be adapted considering many factors, including the type of cancer, type of oncologic treatment, COVID-19 incidence on the location, and availability of health care facilities.

A concern in low-middle income countries (LMICs) is the treatment of cervical cancer. Cervical cancer is the fourth most incident cancer and the fourth cause of cancer death among women, with 85% of the cases occurring in LMICs [5]. In these countries, the availability of radiotherapy equipment, which is essential for cervical cancer treatment, is frequently insufficient, leading to the need to rationalise its use [6, 7]. When facing the COVID-19 pandemic, this need for rationalisation increases. In LMICs, access to COVID-19 tests is also lower than in high-income countries and represents an additional challenge [8].

Another important particularity is that most cervical cancer cases are diagnosed in young women (median age of 50 years) and as localised potentially curable disease [9]. Despite the relevant impact of cervical cancer, scientific research and discussions by international societies are scarce due to the low frequency of this neoplasia in places as Europe and the United States of America.

In this paper, we aim to discuss the cervical cancer scenario during the COVID-19 pandemic and provide specialists recommendations for its management in LMICs.

## General recommendations

In addition to all the measures recommended to the overall population in terms of social distancing, hand hygiene and education on COVID-19 infection symptoms, some recommendations can be made for cancer patients in general. First of all, it is important to highlight that any treatment decision should be based on a case-by-case analysis, which should balance the risks associated with treatment delay or discontinuation versus the risks of COVID-19 exposure and infection.

Building lasting recommendations for all the cases is almost impossible. The complexity of patients and disease varies in different scenarios. In the decision-making process, it is also important to consider the working conditions of health professionals’ teams and the availability of resources. The isolation measures of medical staff, restrictions on face-to-face meetings and losses of professionals affected by Covid19 are additional difficulties. Given this situation, the maintenance of a virtual tumour board is a measure that can be very useful. Different existing communication platforms can be used, providing possibilities for discussing cases with the participation of a multidisciplinary team.

Whenever possible, the treatment should be done in the outpatient setting, avoiding unnecessary hospitalisations. This strategy helps to minimise the risk of patient exposure to the COVID-19 virus and decreases the demand for health care services.

Patients who attend to cancer care facilities should be screened for COVID-19 symptoms. In the case of COVID-19 suspicion, they should be ideally transferred to units focused on COVID-19 care. Additionally, the number of patients’ companions for clinic visits should be limited to one person at most. Visits to hospitalised patients should be restricted as well and visitors should also be screened for COVID-19 symptoms.

In terms of oncologic treatment during the COVID-19 pandemic, anti-cancer treatments have been associated with increased risk of severe events as already mentioned [3]. Considering this, treatment interruptions should be considered for the patients with active COVID-19 infection until patient recovery with resolution of symptoms, especially in cases of immunosuppressive treatments such as cytotoxic chemotherapy [10]. Despite the low availability of COVID-19 tests in LMIC, we highly recommend testing patients who are currently receiving oncologic treatments since test results will guide treatment decisions.

Active COVID-19 infection should be determined by the presence of symptoms associated with a positive reverse transcription-polymerase chain reaction (RT-PCR) assay for SARS-CoV-2 from an upper respiratory sample [11]. Since false-negative results occur frequently with RT-PCR assay, especially in the first days of the disease, this test should be repeated if initially negative and the presumptive diagnosis of COVID-19 infection based on characteristic findings of chest computed tomography is also acceptable [12, 13].

For cancer patients without COVID-19 infection, the start or continuation of treatment should be evaluated individually. In cases of advanced incurable cancer that has been treated with systemic therapy with satisfactory disease control, treatment pauses can be considered during the pandemic period. On the other hand, if a procedure delay may impact negatively patient’s health, an effort should be made to avoid this delay, as recommended by the Society of Gynecologic Oncology [14, 15].

## Priorities in cervical cancer management

Treatment for localised potentially curable cervical cancer (stages I-IVA) should be considered a cancer treatment priority. Thus, as long as local conditions allow it, definitive treatments should be started and continued. Most patients with this diagnosis have less than 60 years, representing a group with great life expectancy after successful curative treatment [16].

For patients with early-stage cervical cancer, both surgery and radiation therapy are acceptable treatment strategies. To decide between the two treatment options during the COVID-19 pandemic, local conditions of the health systems should be considered. Although surgery has the disadvantage of requiring patient hospitalisation, it allows the conclusion of treatment in a single moment. If required by local conditions, a surgical procedure delay of 4–8 weeks would be acceptable in this situation [17, 18].

Radiation therapy, otherwise, requires multiple daily visits to the health care facility. During this period in which individuals’ dislocations are restricted, this may represent a major challenge. Especially in LMIC, radiation therapy facilities are not largely available and are localised in a few reference centres, which difficult importantly patient access during the COVID-19 pandemic. In the face of this, the surgery for early-stage cervical cancer may be a more suitable option in many locations.

For locally advanced cervical cancer, the standard treatment is definitive chemoradiation. Once again, since this treatment is potentially curative, it should remain a priority. Previous studies have shown that delays to initiate chemoradiation after diagnosis of locally advanced cervical cancer and duration greater than 8 weeks to conclude the therapy are both associated with poorer overall survival [19–21]. Thus, an early chemoradiation therapy, ideally without interruptions, should continue to be pursued. To decrease the number of visits to the health care facility, hypofractionated radiation therapy could be discussed in selected cases [17].

Of note, in many situations, the oncologic treatment may represent an urgency rather than an elective procedure. This is the case of patients who presents with complications related to cancer, such as bleeding, which requires immediate measures.

Finally, for patients with metastatic cervical cancer, first-line chemotherapy (with or without bevacizumab, according to availability) should also be considered as a priority treatment. This therapy is associated with a clear survival benefit, justifying its continuation as long as local conditions allow it [22, 23].

## Non-priorities in cervical cancer management

Oncotic colpocytology (Pap smear) is a valuable screening tool, allowing the identification and treatment of premalignant lesions and early cervical cancer. Nevertheless, postponing Pap smear during the COVID-19 pandemic is an acceptable strategy to minimise contact of individuals with health care units. Additionally, the treatment of intraepithelial neoplasia may be postponed [24].

Decreasing health services burden and preserving its resources is essential. The postponement of elective screening procedures is also a recommendation of the American Society of Clinical Oncology [10].

Moreover, as another strategy to decrease health services burden, surgical staging for locally advanced cervical cancer should be avoided. In a randomised trial with 255 patients, no statistically significant difference in overall survival was observed with surgical staging in comparison with standard clinical/radiological staging [25].

Systemic treatment for metastatic cervical cancer after progression on first-line chemotherapy is not a priority in the time being. Currently, no treatment in subsequent lines has been shown to improve overall survival in comparison with best supportive care [26]. Due to this lack of survival benefit and the risks of an immunosuppressive agent during the COVID-19 pandemic, the use of second or later lines of therapy is discouraged.

In other types of cancer, such as breast cancer, the use of neoadjuvant chemotherapy has been suggested during the COVID-19 pandemic as a strategy to delay surgical treatment [27]. In cervical cancer, however, no clear benefit of neoadjuvant chemotherapy before chemoradiation has been shown. Additionally, a randomised phase II study suggested a potentially detrimental effect on the use of neoadjuvant chemotherapy [28]. Considering this, we do not recommend the use of neoadjuvant chemotherapy to postpone the definitive chemoradiation for locally advanced cervical cancer, unless it is used in a clinical trial context.

For patients who have been successfully treated with curative therapy and are currently in follow-up, clinic visits should be postponed for the maximum interval acceptable if the patient is asymptomatic [10]. As an alternative, telemedicine should be considered where available for the follow-up visits [10, 29].

**Figure 1.**
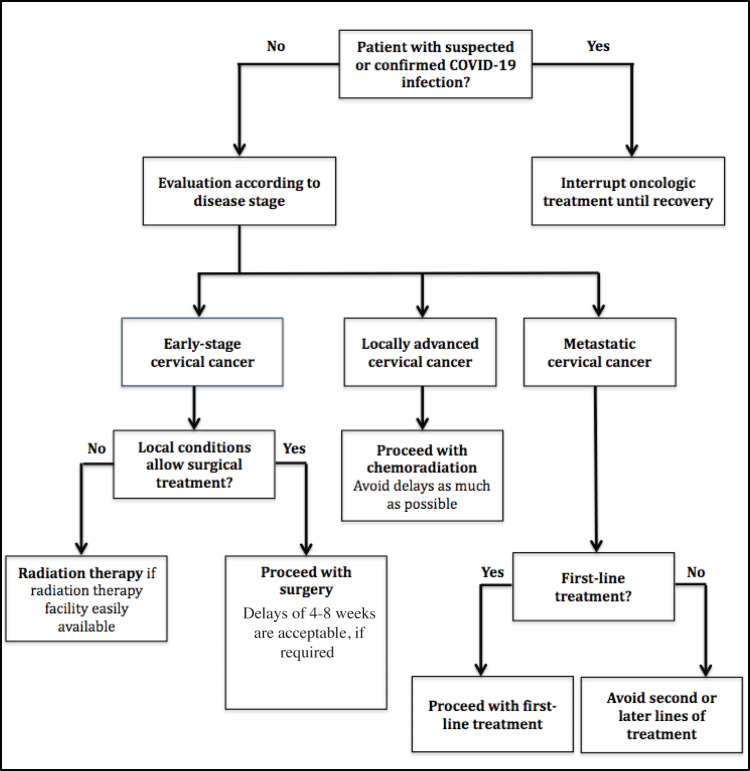
Flowchart of recommendations for the management of cervical cancer patients in active treatment during the COVID-19 pandemic.

A summary of the recommendations for cervical cancer management during the COVID-19 pandemic is shown in Figure 1 and Table 1.

## Conclusions

The world faces a uniquely challenging moment with the COVID-19 pandemic. Significant adaptation of health care services has been required to assist the COVID-19 patients, at the same time as continuing to assist other patients who cannot have their treatments postponed. During this crisis, careful attention is required for some high-risk groups such as cancer patients.

Cervical cancer patients are a particularly delicate group due to patients’ young ages and the potentially curative disease for the majority of cases, occurring mainly in LMICs. We provided a series of recommendations for the management of these patients during the COVID-19 pandemic, especially focused on LMICs. Although oncology societies have provided helpful general recommendations for cervical cancer management, recommendations based on the health services particularities in these countries were lacking.

Finally, we highlight that the management for each patient should be decided on a case-by-case basis, balancing the risks and benefits of each strategy during this period.

## Authors’ contributions

All authors participated in the study conception and design, literature search and data collection and interpretation.

Maria del Pilar Estevez-Diz and Renata Colombo Bonadio participated in the manuscript writing and construction of tables and figures.

All authors reviewed the manuscript and approved the final version.

All authors are accountable for all aspects of the work.

## Disclosures/conflicts of interest

Renata Colombo Bonadio has received financial support for educational programs from AstraZeneca and financial support for attending symposia from Roche. Vanessa Costa Miranda has received honoraria from Mundipharma. All other authors have no disclosures/conflicts of interest.

## Funding

No funding to declare.

## Figures and Tables

**Table 1.** Recommendations on priority and non-priority procedures for cervical cancer management during COVID-19 pandemic.

Priority	Non-priority
Surgery for early-stage cervical cancer—consider deferring until 4–8 weeks in regions with high COVID-19 risk. Radiation therapy is an acceptable altern‑ative in case of easy access to a radiation therapy facility.	Oncotic colpocytology for cervical cancer screening—can be postponed to preserve health care resources and minimise contact of an individual with health care units
Chemoradiation for locally advanced cervical cancer—delays for treatment initiation and conclusion have a negative impact on overall survival.	Systemic therapy after progression on first-line for metastatic cervical cancer—no overall survival benefit
First-line chemotherapy (with or without bevacizumab, according to availability) for patients with metastatic cervical cancer.	Neoadjuvant chemotherapy before chemoradiation for localised cervical cancer—should be avoided due to the lack of a clear benefit and the possibility of a detrimental effect.
Surgical or non-surgical procedures to treat urgent complications (e.g., bleeding) in patients with a potentially curative disease.	Follow-up visits after curative treatment—in case of asymptomatic patients, clinic visits can be postponed or replaced for telemedicine
